# Society Notices

**Published:** 1901-12

**Authors:** Wm. H. Gribble

**Affiliations:** Secretary


### SOCIETY NOTICES.

The annual session of the Ohio State Veterinary Association will be held at the State University, January 14 and 15, 1902. Several papers of interest will be read, and the clinics of last year having proven a success, it has been decided to even improve upon them, if possible. Every graduate veterinarian is invited to attend, and if not a member, but is eligible, he may become one.

The fact that the University contains a well-equipped veterinary college should interest every Ohio veterinarian to desire to become acquainted with it, and this meeting affords him an excellent opportunity. Come and get acquainted, and you will go home not knowing any less and probably a great deal more.

WM. H. GRIBBLE,
Secretary.

---

